# AI bot to detect fake COVID‐19 vaccine certificate

**DOI:** 10.1049/ise2.12063

**Published:** 2022-05-11

**Authors:** Muhammad Arif, Shermin Shamsudheen, F Ajesh, Guojun Wang, Jianer Chen

**Affiliations:** ^1^ School of Computer Science Guangzhou University Guangzhou China; ^2^ Faculty of Computer Science and Information Technology, Jazan University Jazan Saudi Arabia; ^3^ Department of Computer Science and Engineering Sree Buddha College of Engineering Alappuzha India

**Keywords:** artificial intelligence, COVID‐19, deep learning, forged certificate, vaccine certificate

## Abstract

As the world is now fighting against rampant virus COVID‐19, the development of vaccines on a large scale and making it reach millions of people to be immunised has become quintessential. So far 40.9% of the world got vaccinated. Still, there are more to get vaccinated. Those who got vaccinated have the chance of getting the vaccine certificate as proof to move, work, etc., based on their daily requirements. But others create their own forged vaccine certificate using advanced software and digital tools which will create complex problems where we cannot distinguish between real and fake vaccine certificates. Also, it will create immense pressure on the government and as well as healthcare workers as they have been trying to save people from day 1, but parallelly people who have fake vaccine certificates roam around even if they are COVID/Non‐COVID patients. So, to avoid this huge problem, this paper focuses on detecting fake vaccine certificates using a bot powered by Artificial Intelligence and neurologically powered by Deep Learning in which the following are the stages: a) Data Collection, b) Preprocessing to remove noise from the data, and convert to grayscale and normalised, c) Error level analysis, d) Texture‐based feature extraction for extracting logo, symbol and for the signature we extract Crest‐Trough parameter, and e) Classification using DenseNet201 and thereby giving the results as fake/real certificate. The evaluation of the model is taken over performance measures like accuracy, specificity, sensitivity, detection rate, recall, f1‐score, and computation time over state‐of‐art models such as SVM, RNN, VGG16, Alexnet, and CNN in which the proposed model (D201‐LBP) outperforms with an accuracy of 0.94.

## 1 INTRODUCTION

Covid‐19 is an extremely contagious pandemic caused by some novel coronavirus (SARS‐CoV‐2) that initially emerged around late December 2019 in Wuhan, Hubei Province, China. The World Health Organization (WHO) disclosed the COVID‐19 outbreak on 12 March 2020 [1]. Researchers and politicians were striving around the clock, 7 days a week, to identify remedies as well as to develop plans to control the pandemic and reduce their effects on human life and the economy [2]. SARS‐CoV‐2 has been mostly propagated to people via respiratory droplets (sneezing, coughing, and talking) and contaminated surfaces [3, 4]. SARS‐ CoV‐2's notable feature is its ability to remain on a wide range of surfaces for up to 9 days at ambient temperature, allowing for faster transmission [5]. This virus can induce Acute Respiratory Distress Syndrome (ARDS) or multi‐organ malfunction that can lead to a person's physiological deterioration and death [5, 6, 7].

Throughout this worldwide health calamity, the healthcare field has been seeking innovative tools to manage and prevent the outbreak of COVID‐19 (Coronavirus) epidemics. However, artificial intelligence (AI) is a tool that may readily trace a virus's transmission, detect high individuals, as well as assist in real‐time outbreak control. It is also able to forecast mortality danger by thoroughly analysing the individuals' earlier data. Artificial intelligence can help in the battle against it by offering population screening, clinical assistance, notification, as well as infection control comments [8, 9, 10, 11]. As an evidence‐based medical tool, the above technique does have the capacity to boost a COVID‐19 patient's planning, treatment, as well as reported outcomes. The following are some of the applications:

- Early illness identification and diagnostics
- Treatment management
- Personal contact tracing
- Case and mortality forecasts
- Drug and vaccine innovation
- Decreasing the amount of labour that healthcare personnel have to do
- The disease's prevention

**FIGURE 1:**
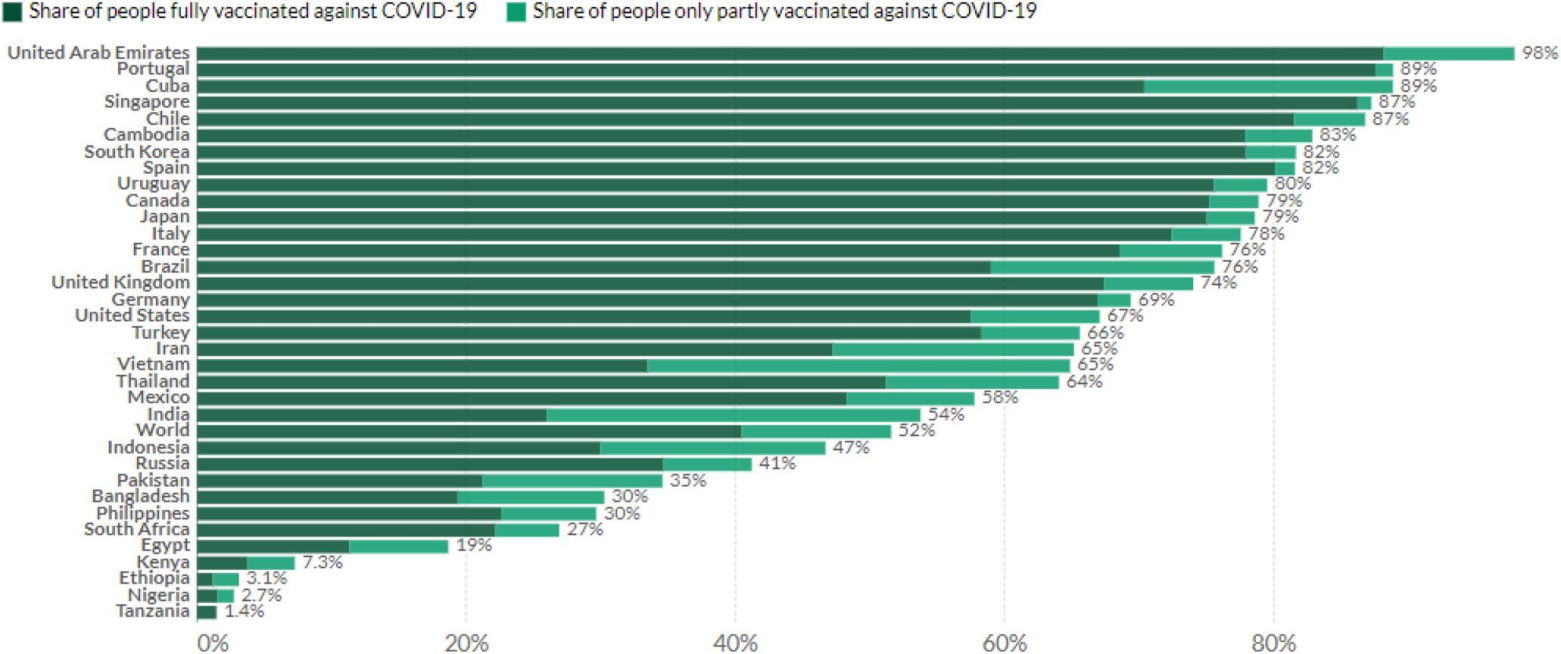
The overall percentage of vaccinated people around the world [12]

Based on Our World in Data, 51.6% of the worldwide population has had at least one dosage of the COVID‐19 immunisation. 7.45 billion doses were distributed worldwide, having 31.1 million doses provided per day. In lesser‐income countries, only 4.5% have got at least one dose. Figure 1 describes statistics on the percentage of people who have been vaccinated in the world until 12 November 2021 [13].

Government and healthcare workers are on the verge of reaching out vaccines to every country around the world to make everyone immunised from this rampant virus. In recent days, many countries have imparted the rule to show the vaccination certificate as a proof of immunisation for travelling or entering into premises of commercial and noncommercial organisations. So fake vaccination is a bigger concern than ever. Some people even depend on social media platform to get fake vaccination certificates. To address this issue, this paper focuses on an application of AI where a bot is used for determining the forgery of vaccination certificates.

### 1.1 Key objectives

This paper focuses on bringing an effective AI application for detecting the forgery of vaccine certificates for which the following are the objectives:

- AI‐powered bot for detecting the fake COVID‐19 vaccine certificates.
- Preprocessing these certificates for noise removal.
- Extracting quintessential features from certificates such as logo, symbol, words, and crest‐rough parameter for signature and seal using Local Binary Pattern.
- Detection using Densenet201 for fake/real vaccine certificate.
- This DL algorithm is trained for an AI bot where people can upload their certificate and the result will detect a valid or invalid certificate.

***Organisation of this paper***—Section 1 explains the introductory area of AI and COVID‐19, Section 2 depicts the relevant efforts proposed in these areas, Section 3 represents the technique for establishing one such bot, Section 4 demonstrates performance assessment and concludes with the details in Section 5.

## 2 RELATED WORKS

Eduard et al. (2020) [14] investigated the use of machine learning‐based technologies for eliminating counterfeit items. Machine learning‐based image and text identification and categorisation can be a crucial tool in the battle against counterfeiting. End‐users may identify counterfeits accurately and rapidly by comparing them to trained models using automatic picture and text recognition, as well as categorisation of product information. The intention of such research is often to build up simple applications in which the end‐user can identify counterfeit products and help combat product piracy.

To computerise forged news detection in Twitter datasets, Abdullah‐All‐Tanvir et al. (2019) [15] suggested a method for spotting falsified media texts from Twitter postings by working out whether to foresee highly precise evaluations. After that we compared 5 familiar Machine Learning techniques, including Support Vector Machine, Naive Bayes Approach, Logistic Regression, as well as Recurrent Neural Network approaches, to show how efficient the categorisation results upon this dataset were. SVM and Nave Bayes classifiers outperformed the other methods in our experiments.

Kadam et al. (2020) [16] demonstrate systematic mappings of current literature for image forgery identification employing deep learning and explainable AI in Kadam et al. (2020) [16]. For data analysis, Sciencescape, Gephi, Tableau, and VOS Viewer were used, as well as the Scopus database. The investigation showed that the vast majority of reviews on image forgery detection via deep learning and explainable AI had only recently been investigated. The research investigations centred on this research subject have found that universities/institutions in the United States are at the forefront.

Pomari et al. (2018) [17] described a unique strategy for recognising pictorial splicing which incorporates a strong representation strength of Illuminant Maps with Convolutional Neural Networks as the manner of studying the most essential clues of a counterfeit straight from existing training data. This paper offers an approach that bypasses the time‐consuming feature engineering process, allows for the detection of counterfeit regions, as well as acquires a classifier precision of more than 96%, beating state‐of‐the‐art strategies over a massive range of datasets. Analysing some dubious real‐world pictures that recently made the news significantly highlights the proposed method's potential applications.

Mouratidis et al. (2020) [18] devised a new strategy for an automatic recognition of falsified reports on Twitter which incorporates (a) pairwise input text, (b) a unique deep neural network learning structure that enables adaptable input merging at multiple network layers, as well as (c) a variety of input mechanisms, such as word embeddings as well as both linguistic and network account character traits. Moreover, tweets were divided into two categories: news headlines and news content, as well as a large testing procedure, which uses both to accomplish classification tests. Our major findings reveal a high level of overall accuracy in detecting fake news. While utilising fewer characteristics as well as embeddings out from tweets, the suggested deep learning structure beats state‐of‐the‐art classifiers.

Amel et al. (2016) [19] have used the Dresden database to train a CNN to solve this categorisation task. Preprocessing with a specially developed 2D high‐pass filter was also a part of their work. Their total accuracy, although, falls short of the state‐of‐the‐art accuracy outlined in ref. [15]. Eric Kee et al. [20] introduces the notion of a Content Adaptive Fusion Network that is essentially a group of CNNs with variable kernel sizes, to categorise camera brands and devices with a modest accuracy of around 95%. Despite the vast amount of research done in this area, the recognition of camera models from post‐processed photos has received minimal focus (including distinct JPEG Compression Rate, Resized, Gamma‐Corrected pictures, etc.) While several of these examples have been studied separately, few have attempted to combine them into a single framework. Image authentication using JPEG headers [21], picture fraud identification employing intrinsic statistical fingerprints of pictures [22], identifying doubly compressed Pictures utilising Discrete Cosine Transform (DCT) [23], as well as the most latest utilization CNN to identify picture manipulation in ref. [24] were also just a few instances of efforts made in identifying and categorising image manipulations using this approach.

## 3 METHODOLOGY

**FIGURE 2:**
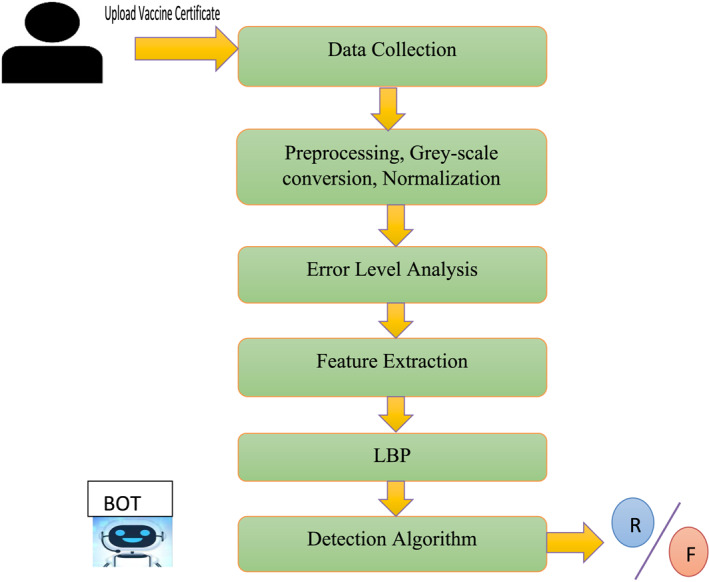
Overall proposed framework

Figure 2 depicts the proposed framework's overall structure, with the stages listed below: a) Data Collection where we collected around 5000 vaccine certificates over different countries, b) Preprocessing of these images happens when we initially convert those into grey‐scale and then perform normalisation, c) On preserving pictures often at a given level of quality then determining a disparity out from compression level, error level analysis can be used to identify images that have been modified, d) Feature extraction where LBP is used for extracting texture features like logo, symbol, words and Crest‐Trough parameter for signature and seal, and e) Classification where both the extracted features are passed to this stage where Densenet201 perform the detection using testing an training data and thereby giving binary classified result.

### 3.1 Data Collection

For any model to be worked, proper feeding of data is important in which here for our deep learning framework to be performed, a sufficient amount of data is collected where here we feed vaccine certificates images which have been collected from various countries like India, the US, UK, Australia, Dubai, China, and Japan. Every vaccine certificate image is of the size 2400 × 3500 px and the dataset has forged as well as real certificates. Here 70% of the dataset (3000) was used for training and the remaining 30% (2000) for testing purposes.

### 3.2 Preprocessing

This is the next stage where collected images are being preprocessed for removing noise for better processing. So here we perform grey‐scale conversion and the normalisation for normalising these images.

***Grey‐scale conversion***: Intensity, greyscale, or grey level images are images that only involve intensity. One of the most basic images enhancing methods is grayscale conversion. Grayscale models were typically utilised to get descriptors rather than simply working on colour pictures because it simplifies the approach as well as lowers computational demands. Indeed, in numerous applications, colour may be of minimal use, and incorporating extraneous information may enhance the amount of training data needed to attain high performance, as is the case with text recognition and identification. Many algorithms have been proposed for grayscale conversion. It has been demonstrated that not all colour‐to‐grayscale algorithms function equally well [25] and that the Luminance method outperforms other alternatives for texture‐based image processing [25]. In our situation, we employ the Luminance algorithm, which uses a component‐wise weighted mixture of the RGB channels to match human brightness perception. Luminance is by far more important in distinguishing visual features [26]. Many algorithms exploit this property for jpeg compression, where images are compressed within YCbCr colour space, and chrominance (Cb, Cr) is quantised and compressed more than luminance (Y) [27].

Luminance=0.3R+0.59G+0.11B

**FIGURE 3:**
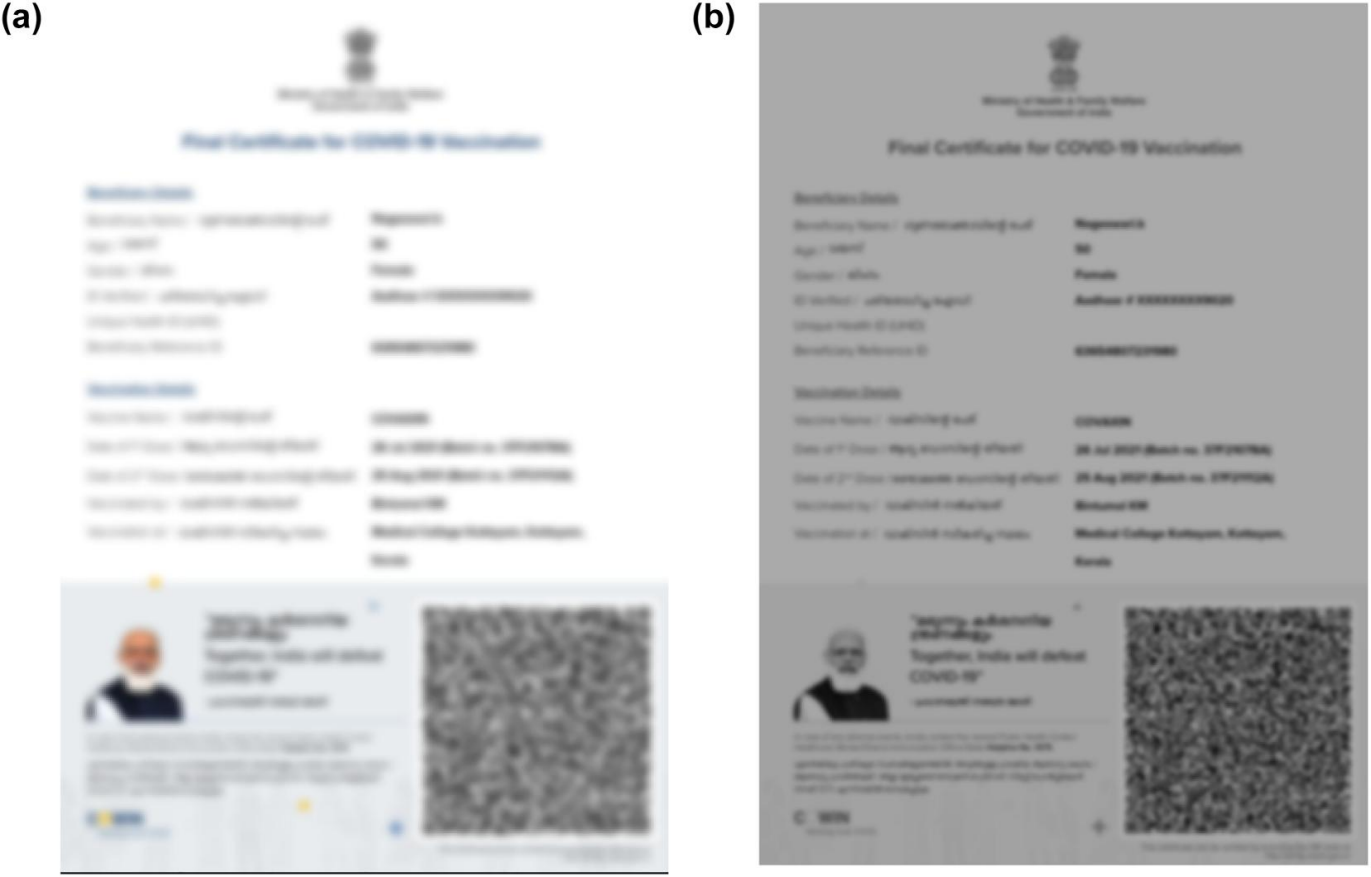
(a) Original Image of the certificate and (b) Grey‐scale conversion

R stands for the red value, the green value is *G*, and blue value is denoted by the letter B. Figure 3a,b depicts the instance of the image converted to greyscale conversion.

***Normalisation***: Provided a collection of data *D* = {x (i)} N *i* = 1, a normalisation procedure is a function: Φ: *x* 7−→ xˆ, that guarantees that the converted data Db = {xˆ (i)} N *i* = 1 meets several statistical properties (centring, scaling, standardising, as well as whitening) [12, 28, 29, 30, 31].

The transformation is defined as follows by centring:

xˆ=ΦC(x)=x−ED(x)

This guarantees whether the normalised output *x* does have a zero‐mean attribute, that may be expressed as EDb(*x*) = 0.

Scaling expresses a transition as follows:

xˆ=ΦSC(x)=Λ−12x

In this case, = diag (2 1,..., 2 d), in which σ 2 *j* is the mean square over sample data for an *i*th dimension: σ 2 *j* = ED (*x* 2 *j*). Scaling assures that a normalised output *x* have unit variance, typically expressed by EDb (xˆ 2 *j*) = 1 for all *j* = 1, ..., *d*.

Standardising seems to be a composition procedure that integrates centring as well as scaling, as demonstrated by:

xˆ=ΦST(x)=Λ−12(x−ED(x))

Standardisation assures that certain normalised output *x* does have a zero‐mean as well as a unit variance.

Whitening defines a change as follows:

xˆ=ΦW(x)=Λ˜−12Dx

where Λ = ˜ diag (˜σ1,. . ., σ˜d) as well as *D* = [d1,..., dd] be the eigenvalues and corresponding eigenvectors of the covariance matrix. Whitening guarantees that the normalised output xˆ does have a spherical Gaussian distribution, which may be expressed as ED (xˆxˆ *T*) = I.

### 3.3 Error level analysis

**FIGURE 4:**
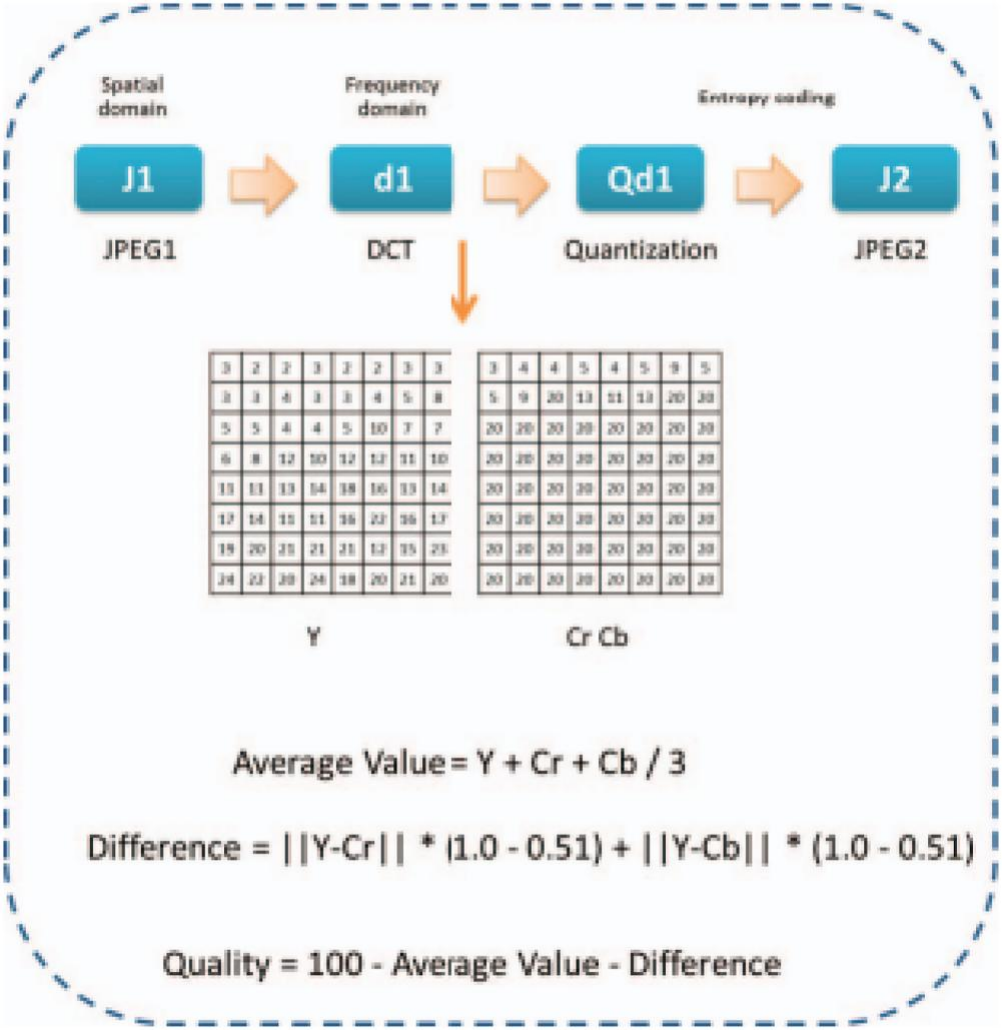
ELA approximation flow

ELA is utilised for determining the parts of a picture with varying compression rates. A JPEG picture should have an identical overall image quality [32, 33, 34]. If a portion of the image has a significantly varying error rate, this might suggest a digital change. A JPEG picture may be resaved 64 times on average with no apparent changes unless the image is updated. The considerable difference in error level of a JPEG picture indicates image counterfeiting when employing ELA. The downside of ELA is that it provides a misleading identification when it comes to images with low JPEG quality and considerable re‐colouring. The quantization table quantises the frequency coefficient from the picture, which is then followed by the entropy encoding procedure. The approximation technique for ELA is depicted in Figure 4, and the quality of image computation may be characterised by calculating the distinction among the average value out from quantization table Y (luminance) as well as CrCb (chrominance).

Utilising ELA, a picture is subdivided into 8 × 8 pieces as well as recompressed independently with the specified error rate, like 95%. If the picture is unaltered, each square should be assigned the same quality rating. The error level refers to the loss of data that occurs when a picture is stored in the necessary format. Furthermore, during resave procedures, the degree of inaccuracy would be enhanced. Eventual resave procedures might lower the error level potential, resulting in darker ELA findings. The square grid may achieve its lowest error level after some resave procedures. As a result, each resave process may lose out on frequency and information. Nevertheless, in ELA, the amount of inaccuracy is constrained to 8 × 8 blocks. If the quality level remains unchanged, the block error has attained its local minimum. Whether there is a considerable amount of variation, the pixels were not at their local minima, and the differences are then identified afterwards.

### 3.4 Feature extraction

Feature extraction is indeed a dimensionality reduction approach that lowers a large amount of raw data into more manageable groupings for processing. Because of the huge set of variables within these massive datasets, processing them requires a substantial amount of computational resources. So, in this case, we utilise the Local Binary Pattern to retrieve characteristics such as logos, stamps, and words, and the Crest‐Trough parameter to recover signatures and seals.

Local Binary Pattern is a basic yet effective texture operator that is commonly employed in various computer vision tasks. When it comes to choosing this descriptor, some of the traits which step out will be its resilience to monotonic grey‐level fluctuations, discriminative power, as well as ease of calculation. In ref. [35], Ojala et al. presented the first LBP operator. It entails generating an array comprising labels for picture pixels based on the distinction in pixel levels of intensity in a predetermined local neighbourhood focussed on all of them. The descriptor obtains grey‐scale invariance while still capturing small‐scale appearance aspects of an area surrounding it in this approach. It aids in the representation of local geometry features and forms. The computation of LBP is divided into two parts. The LBP transform is computed first. Second, in the final histogram form, the calculated values are grouped. As a result, the general formula for computing the LBP for a pixel gc will be as follows:

$${\text{LBP}}_{P,R,t}=\sum_{p=0}^{P-1}s(g_{p}-g_{c})\times 2^{P}$$

where *P* is the set of symmetrically and evenly distributed sample pixels gp within a circular neighbourhood all over gc of radius R. Function *s* is a binary function that represents local intensity variations. When the circular arrangement of a pattern does have a limited amount of transformations, the phrase uniform relates to the appearance's uniformity. Uniform patterns often account for up to 90% of the overall quantity, and it has been demonstrated how they correlate with discriminating characteristics including edges or key points within the geometric definition. An LBP value is calculated among this instance as follows:

$${\text{LBP}}_{P,R}^{u^{2}}=\left\{\sum_{p=1}^{P-1}s\left(g_{p}-g_{c}\right)\right.\;\text{if}\;U\left({\text{LBP}}_{P,R}\right)\leq 2\;\;\{P+1\;\text{otherwise}\;x\geq P$$

where, s(*x*) = 1 if *x* ≥ 0, and 0 otherwise, and function U is the metric that describes the number of spatial transitions among pixels in a circular shape is as follows:

$$U\left(N_{P,R}\right)=|s\left(g_{p}-1-g_{c}\right)-s\left(g_{0}-g_{c}\right)|+\sum_{p=1}^{P-1}|s\left(g_{p}-g_{c}\right)-s\left(g_{p}-1-g_{c}\right)|$$

As indicated in ref. [36] the number of transformations utilised to define the pattern uniform was fixed as two. A *P* + 1 uniform pattern could exist inside a circularly symmetric neighbour collection comprising *p* pixels. Eq designates the label matching towards many ‘1’ bits within the pattern (0 ‐ > *p*) to each of them, although the non‐uniform patterns were combined beneath miscellaneous label *P* + 1. An appearance of uniform patterns within deterministic textures [35, 37, 38] lends support to their selection.

Crest trough is the most important parameter of the feature extraction for signature and seal parameters since this consumes remark of all the shapes of a signature. All signature crest, as well as trough points, follow a consistent pattern [39]. Consider a 3 × 3 matrix with black components, hence the top‐right Associate in Nursing top‐left corner black components can have the nearby “Black pixel:White pixel” quantitative relation five: three, as well as exclusively a specific set of neighbours will be white, black in eight neighbour theory. So when rotation is complete, these spots will represent the curve edges of a crest inside the signature. Trough points are the bottom‐left as well as bottom‐right corner black pixel area units. The distance among every consecutive crest as well as trough positions produces a sum, which is then divided evenly mostly by the realm of a signature to obtain a novel difference specific towards this signature. As a result, fraud detection would be accomplished via retrieving attributes from linked people's authentic signatures then contrasting them to the comparison signature. When the signature is recognised on the individual to whom it lies, the verified picture, and therefore the collection comprising all signatures pictures of the identified subject, is erased. The signature is deemed original if those three retrieved choices have the computed variants. Otherwise, it is regarded cast. Once enough features have been retrieved and passed for detection, Densenet201 is employed.

### 3.5 Detection using Densenet201

Table 1 shows the entire structure of our approach. The following are some of the components of our model:

- Initially, we choose patches with a size of 256 × 256 from the produced pictures depending on their quality.
- We utilise the extracted patches to coach Dense Convolutional Networks (DenseNets), especially the DenseNet‐201 framework, having patches of size 256 × 256.
- Then, we retrieve features out of second to a final layer for size 256 × 256 so most non‐overlapping patches of size 128 × 128 as well as 64 × 64 out of every training picture utilising the DenseNet‐201 trained on 256 × 256 patches alone. As a result, we effectively have three feature vectors for three distinct patch sizes.
- We next combine all feature vectors generated via this network as well as utilise these to train a secondary network comprised of a Squeeze‐and‐Excitation (SE) block and also a categorisation block. The SE block's outcomes are given into a categorisation block.
- While testing, we build feature vectors in the same way for every 256 × 256 patches, utilising the DenseNet‐201 trained on just 256 × 256 patches. All those characteristics were combined as well as sent to a secondary network, which produces an ultimate forecast for the full picture.

**TABLE 1:** Architecture of Densnet201

Hidden layers	Output size	Densnet‐201
Convolution	112 × 112	7 × 7 conv, stride 2
Pooling	56 × 56	3 × 3 max pool, stride 2
Dense block 1	56 × 56	[1 × 1 conv, 3 × 3 conv ] x6
Transition layer 1	56 × 56	1 × 1 conv
	28 × 28	2 × 2 average pool, stride 2
Dense block 2	28 × 28	[1 × 1 conv, 3 × 3 conv] x 12
Transition layer 2	28 × 28	1 × 1 conv
	14 × 14	2 × 2 average pool, stride 2
Dense block 3	14 × 14	[1 × 1 conv, 3 × 3 conv] x64
Transition layer 3	14 × 14	1 × 1 conv
	7 × 7	2 × 2 average pool, stride 2
Dense block 4	7 × 7′	[1 × 1 conv, 3 × 3 conv] x48
Classification layer	1 × 1	7 × 7 global average pool
		1000D FC, softmax

We employ the 201‐layer DenseNet model proposed in ref.[40]. It is made up of four thick blocks, all having a growth rate of 32. Among successive thick blocks, transition layers have been employed. There is a convolution layer as well as a max‐pooling layer. The network has no reduction or dropout layers. The resultant feature vector's dimensionality is lowered via Global Average Pooling, and the features are eventually categorised utilising a completely linked layer having Softmax as an activation function. Classification probabilities for every category are returned by this layer.

#### 3.5.1 Squeeze and excitation block

**FIGURE 5:**
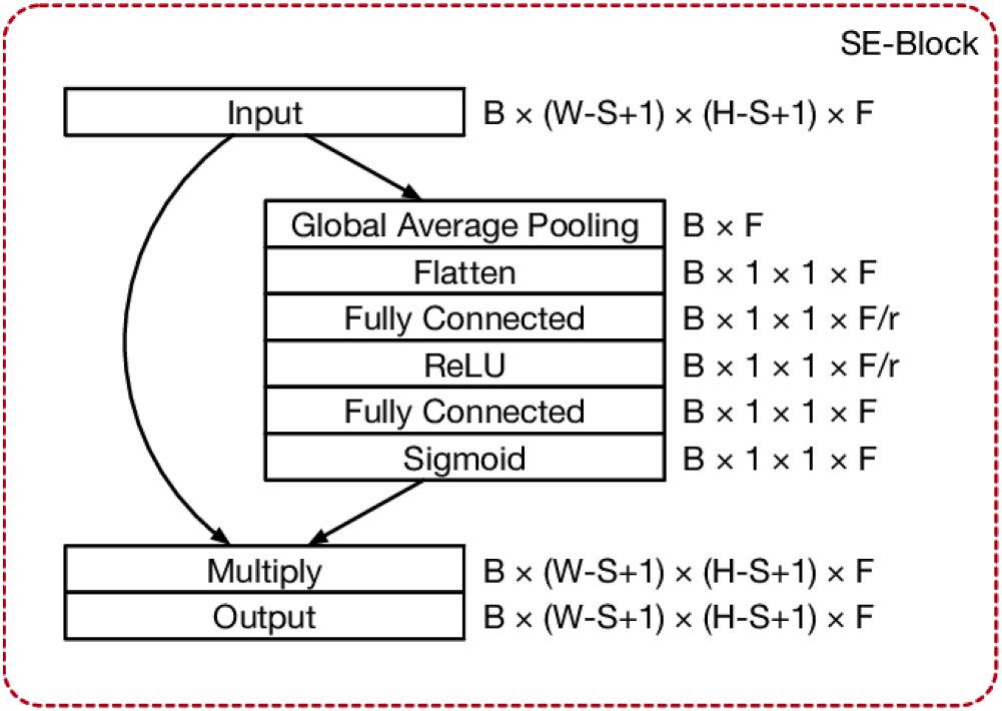
Squeeze‐and‐excitation (SE) block

After the four dense blocks, the output is sent to another unit known as a “Squeeze‐and‐Excitation” (SE) block. Hu, Shen, and Sun [41] were the first to use this component. The goal of this module is to increase a network's representational power by explicitly modelling the interdependencies among its output channels. For doing it, an SE block uses feature recalibration to understand how to use relevant data to preferentially enhance informative aspects while minimising the least valuable ones without affecting the feature vector's dimensions. The interior levels of an SE block, as well as the associated structures, are depicted in Figure 5. As shown below, we build a SE block for doing feature recalibration. These input parameters were squeezed initially to generate the channel descriptor that aggregates all feature mappings across spatial dimensions. This descriptor integrates the worldwide distribution of channel‐wise feature responses, allowing lower levels of the network to use information from the network's global receptive field. Following this is an excitation procedure wherein channel excitation is regulated by sample‐specific activations attained per every medium via a self‐gating process relying upon channel dependence. The characteristic maps are further re‐weighted for providing an SE block output, which is then passed directly to the classification layers [38, 42].

## 4 PERFORMANCE MEASURES

Here the bot is built using python where this bot can be a friendly bot where customers can interact and fulfil their requirements. Here users can upload the certificate and bot functions to tell us whether it is real or fake. On the function block of the bot, we incorporate this Deep learning model which is developed using python in Google Collaboratory, and the hardware specification to build is Intel Core i9‐10,980HKGPU: Nvidia RTX 3080RAM: 32GBStorage: 2 1 TB. The system is analysed utilising characteristics such as accuracy, sensitivity, specificity, recall, f1‐score, detection rate, as well as computation time.

This suggested approach (D201‐LBP) was trained upon 70% of the dataset, and then tested on 30% of the dataset on the network using Stochastic Gradient Descent as the Optimiser about the momentum of 0.9 as well as an initial learning rate of 103, and it is decreasing till the validation loss is reduced. To prevent model overfitting, cross‐validation is employed for the final evaluation of the models. Table 2 depicts the comparative analysis of various models after training and testing. The proposed model (D201‐LBP) outperforms all measures over 3 × 3 and 5 × 5 cross‐validation.

**TABLE 2:** Comparative analysis of state‐of‐art methods

Models	Validation	Accuracy	Sensitivity	Specificity	Recall	F1‐score
SVM		0.76	0.88	0.89	0.92	0.9
RNN		0.82	0.87	0.91	0.94	0.93
VGG16	3 × 3	0.85	0.94	0.95	0.96	0.89
Alexie		0.86	0.97	0.97	0.88	0.82
CNN		0.9	0.98	0.97	0.94	0.95
D201‐LBP (Ours)		0.94	0.98	0.96	0.97	0.97
SVM		0.82	0.85	0.79	0.86	0.94
RNN		0.87	0.88	0.87	0.91	0.93
VGG16	5 × 5	0.88	0.93	0.92	0.94	0.92
Alexnet		0.91	0.92	0.95	0.9	0.92
CNN		0.93	0.95	0.97	0.93	0.96
D201‐LBP (Ours)		0.95	0.98	0.97	0.95	0.97

**FIGURE 6:**
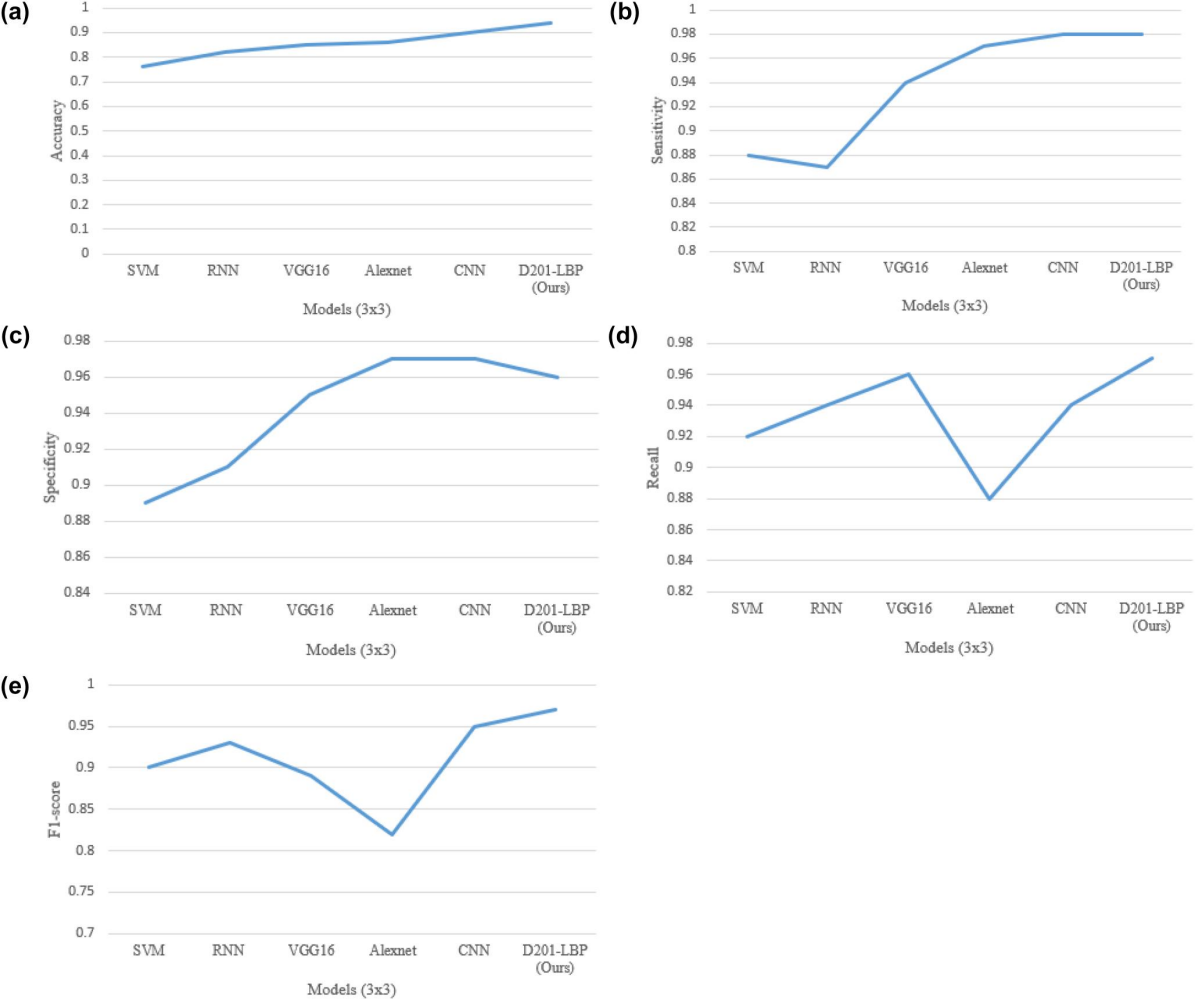
(a) Models Accuracy, (b) Models versus Sensitivity, (c) Models versus Specificity, (d) Models versus Recall, and (e) Models versus F1‐score over 3 × 3 validation

Figure 6a,b,c,d,e depict the graphical representation of various model over various performance measure under 3 × 3 validation in which D201‐LBP brings a clear view with accuracy (0.94) when compared to other models. The model that performs moderately was SVM (0.76) due to its limited structure for detecting such complex images.

**FIGURE 7:**
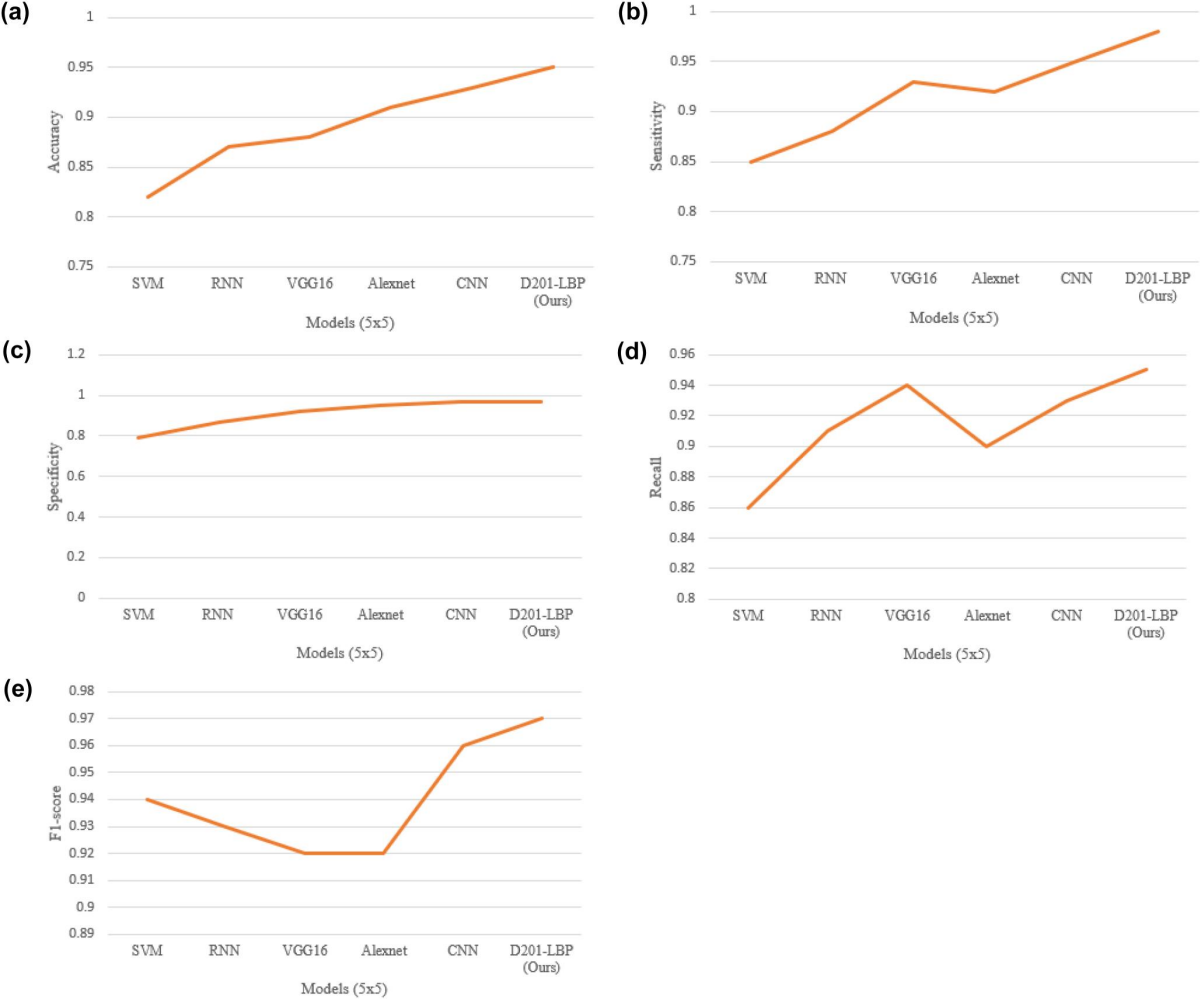
(a) Models Accuracy, (b). Models versus Sensitivity, (c). Models versus Specificity, (d). Models versus Recall, and (e). Models versus F1‐score over 5 × 5 validations

Figure 7a,b,c,d,e depict the graphical representation of various model over various performance measure under 5 × 5 validation in which D201‐LBP brings a clear view with accuracy (0.95) when compared to other models. The model that performs very similarly to densenet201 was CNN due to the handling of complex images with 0.93.

**FIGURE 8:**
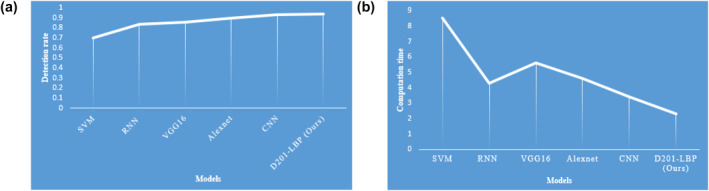
(a) Models versus Detection rate and (b). Models versus CT

Figure 8a,b depicts the graphical representation of the detection rate and computation time of each model in which the proposed model (D201‐LBP) outperforms at detecting with 0.94% and 2.31 computation time.

## 5 CONCLUSION

This paper brings a new aspect of the application of Artificial Intelligence for detecting forged vaccine certificates. With the help of an AI bot, we can function it depending on the requirements using a deep learning algorithm as well as bringing effective results. In this paper, Densnet201 is used for detecting fake vaccine certificates at a greater detection rate contrasted to other state‐of‐art approaches. Also, the use of LBP as a texture feature extraction method boosts the classification rate even more for this kind of a complex process. As there are ample numbers of papers related to the detection of fake news, fake documents, this will be the first and best attempt that brings a new application of AI and also to detect any forgery in vaccine certificates at a greater detection rate. This will be best for understanding the progress of AI and Deep Learning. As this paper mentions the story of detecting forged vaccine certificate, mostly this will be much potentially helpful for the research community for better integration and development of a model for even more accurate results from theoretical and practical aspects. The most advanced deep learning techniques have certain limitations over detecting these certificates. The limitations include handling the enormous volume of data, improving the quality of data for better processing and suitability to application domain. Moreover necessary steps must be taken to ensure security and privacy, as well as directing DL in the appropriate direction by integrating expert knowledge. Constructing DL systems that are time‐sensitive will result in a more effective DL model. Also, there are no currently available datasets for these certificates for a model to be trained and tested, and to implement we have prepared huge data manually.

## CONFLICT OF INTEREST

The authors declare that there are no conflict of interests.

## Data Availability

The data used in this research will be available upon request from the corresponding author.
